# Trends in Extinction Risk for Imperiled Species in Canada

**DOI:** 10.1371/journal.pone.0113118

**Published:** 2014-11-17

**Authors:** Brett Favaro, Danielle C. Claar, Caroline H. Fox, Cameron Freshwater, Jessica J. Holden, Allan Roberts, UVic Research Derby

**Affiliations:** 1 Department of Biology, University of Victoria, Victoria, Canada; 2 Centre for Sustainable Aquatic Resources, Fisheries and Marine Institute of Memorial University of Newfoundland, St John's, Canada; 3 Department of Ocean Sciences, Memorial University of Newfoundland, St. John's, Canada; 4 Department of Geography, University of Victoria, Victoria, Canada; 5 Raincoast Conservation Foundation, Sidney, Canada; 6 Bamfield Marine Sciences Centre, Bamfield East, Canada; 7 School of Environmental Studies, University of Victoria, Victoria, Canada; Instituto de Pesquisas Ecológicas, Brazil

## Abstract

Protecting and promoting recovery of species at risk of extinction is a critical component of biodiversity conservation. In Canada, the Committee on the Status of Endangered Wildlife in Canada (COSEWIC) determines whether species are at risk of extinction or extirpation, and has conducted these assessments since 1977. We examined trends in COSEWIC assessments to identify whether at-risk species that have been assessed more than once tended to improve, remain constant, or deteriorate in status, as a way of assessing the effectiveness of biodiversity conservation in Canada. Of 369 species that met our criteria for examination, 115 deteriorated, 202 remained unchanged, and 52 improved in status. Only 20 species (5.4%) improved to the point where they were ‘not at risk’, and five of those were due to increased sampling efforts rather than an increase in population size. Species outcomes were also dependent on the severity of their initial assessment; for example, 47% of species that were initially listed as special concern deteriorated between assessments. After receiving an at-risk assessment by COSEWIC, a species is considered for listing under the federal Species at Risk Act (SARA), which is the primary national tool that mandates protection for at-risk species. We examined whether SARA-listing was associated with improved COSEWIC assessment outcomes relative to unlisted species. Of 305 species that had multiple assessments and were SARA-listed, 221 were listed at a level that required identification and protection of critical habitat; however, critical habitat was fully identified for only 56 of these species. We suggest that the Canadian government should formally identify and protect critical habitat, as is required by existing legislation. In addition, our finding that at-risk species in Canada rarely recover leads us to recommend that every effort be made to actively prevent species from becoming at-risk in the first place.

## Introduction

Unsustainable exploitation, climate change, ocean acidification and other anthropogenic impacts have resulted in a global extinction rate that is as much as 1000 times the historic background rate [1]–[3]. Given the irreversibility of extinctions, preventing or reversing the continuing decline of at-risk species is a major focus of conservation [4]. Preserving global biodiversity is also considered essential for human well-being and the maintenance of ecosystem processes [3], [5]–[7].

Many countries have legislation that explicitly protects species at risk of extinction. In general, such legislation is designed to identify vulnerable taxa, establish recovery plans, prevent further declines, and promote recovery [8]. Recognizing that habitat loss is the leading cause of extinction [9]–[12], the identification and preservation of habitat is often required, contributing to the stabilization and recovery of threatened species [13]. Despite the implementation of laws and conservation programs, global biodiversity continues to decline [3], [5], [14].

In Canada, species at risk are identified and protected in a multi-step process. The process begins with the Committee on the Status of Endangered Wildlife in Canada (COSEWIC), an independent scientific body formed in 1977. COSEWIC assesses the status of candidate species that are potentially at risk of extinction or extirpation [15]. This body only considers scientific evidence relevant to a species' recovery potential, and ignores socioeconomic costs or benefits of protection [15]. Their assessments are based on the Canadian extent of the species' range, even if the species occurs in the United States or elsewhere [15]. Species assessed as at-risk by COSEWIC do not automatically secure legal protection from the committee's decision. Rather, protection is issued under the Species at Risk Act (SARA), which was passed in 2003, and which formalized the use of COSEWIC assessments as the scientific basis for listing decisions [16]. Upon receipt of a COSEWIC assessment, the Minister of the Environment must issue a response statement indicating whether the species will be listed at the status recommended by the assessment, or whether more information is required [17]. The ultimate decision whether to list may also incorporate the socio-economic impacts of listing. If listed, it becomes illegal to kill or harm individuals of that species and the species' critical habitat must be identified and protected to the extent possible [18]. Protections derived from SARA automatically apply to federal lands (including oceans), but for these measures to apply outside of these areas (e.g. in provincial or private land) complementary protections must be implemented that applies to the species and its habitat [19]. If the Minister of the Environment decides that provincial or territorial law is insufficient to effectively protect a listed species, the federal government has the power to apply SARA's protections to provincial or territorial lands [20]. Recovery strategies and action plans (required by SARA) are mandated to be released within a specified timeframe, the length of which depends on the species' designation and when it was listed [21], [22]. Furthermore, SARA requires that management plans be produced for species that are not at immediate risk of extinction or extirpation, but are nevertheless of ‘special concern’ since they may become at-risk in the future [23]. Species of special concern do not receive the full protections offered to species listed under SARA as threatened, endangered, or extirpated.

Previous studies have identified biases, limitations, and a general lack of implementation associated with at-risk species legislation in Canada (Table 1). For instance, harvested species and those found in northern territories are less likely to be listed under SARA [24], [26], as are species threatened by biological resource use, including those that are unintentionally harvested [27]. In addition, recovery strategies and action plans are often not completed within deadlines established by SARA [22], leading to concerns that species are not receiving timely protections, despite being listed. This finding was confirmed in a recent federal court decision [28]. Moreover, COSEWIC itself has been criticized for its assessment criteria and apparent biases [31]–[32]. For example, species with low information availability tend to receive less severe assessments than would be suggested under the precautionary principle [30].

**Table 1:** Summary of review papers related to endangered species assessment and legislation in Canada.

Publication	Reference #	Primary findings
Vanderzwaag and Hutchings (2005)	[51]	Review of SARA implementation related to marine fish. Paper advocates for biodiversity preservation by implementing marine protected areas and modernizing Fisheries Act.
Mooers et al. (2007)	[52]	First identification of taxonomic and regional biases in SARA listing. Northern species and marine fish and terrestrial mammals unlikely to receive SARA-listing.
Findlay et al. (2009)	[24]	Commercially harvested species, species managed by DFO, and species that occur entirely within Canada are less likely to receive listing.
Lukey et al. (2009)	[29]	Changes in assessment status from ‘endangered’ to ‘threatened’ often occur without sufficient justification for the change. Assessment criteria are not always applied consistently.
Lukey et al. (2010)	[30]	COSEWIC assessments do not follow the precautionary principle – lack of information is associated with assessments of species to lower risk categories.
Mooers et al. (2010)	[25]	Most SARA-listed species lack recovery plans. Scientific advice is insufficiently reflected in conservation policy for at-risk species. First review of changes in COSEWIC status across assessments.
Powles (2011)	[31]	General overview of marine fish assessed by COSEWIC.
Taylor and Pinkus (2013)	[53]	Only 17% of recovery strategies led by DFO included critical habitat, as opposed to 63% for those led by Environment Canada. Recovery strategies written after court judgments related to SARA were more likely to identify critical habitat.
Waples et al. (2013)	[8]	Comparison of United States' Endangered Species Act (ESA) with SARA. ESA should adopt a single national scientific body, while SARA should adopt strict deadlines for listing action. The emphasis on socioeconomic factors should also be reduced.
McCune et al. (2013)	[27]	Human disturbance, invasive species, residential development, and ultimately loss of habitat are major threats to the majority of SARA-listed species. Threats differ by taxonomic grouping.
Schultz et al. (2013)	[26]	Imperiled marine fish are unlikely to receive SARA listing if the forecasted cost of listing exceeds $90,000 per decade. The threshold for freshwater fish is $5,000,000. Rationale used in the decision to list was inconsistent between freshwater and marine fish.
Auditor General of Canada (2013)	[22]	All three government agencies involved with species at risk (Environment Canada, Fisheries and Oceans Canada, and Parks Canada) are not meeting obligations to complete recovery strategies, action plans, or management plans. Only seven of 97 required action plans were complete.

In this study, we examine trends in the status of species repeatedly assessed by COSEWIC, used here as a proxy for Canada's effectiveness in species conservation. Using records obtained from COSEWIC [33] and the SARA Public Registry [34], we analyze trends in the designations of species with two or more assessments to determine whether species are, on average, improving, deteriorating, or remaining stable in status. In addition, we examine outcome differences across taxonomic groups, and whether the basic obligation to identify critical habitat for listed species has been met. Finally, we assess whether species listed for longer have better outcomes. While this study builds on previous work reviewing the process of listing species under SARA (Table 1), it is the first to assess the overall trends in status of at-risk species that have been assessed more than once by COSEWIC.

## Methods

### COSEWIC Wildlife Species Database

Species, subspecies, and populations (hereafter ‘species’) that are assessed by COSEWIC are placed into one of five categories on a scale of increasing risk of extinction or extirpation [32]: not at risk, special concern, threatened, endangered, or extinct or extirpated (hereafter extirpated). A sixth category, data deficient, is used when there are insufficient data to classify a species. The criteria for categorization depends on a variety of factors including changes in total numbers of mature individuals, whether a species has a small and declining population size, and the changes in size of its range [35]. COSEWIC's assessment criteria were updated in 2001 [35]. Although the same assessment categories were employed after the revision in 2001, species required a more severe decline in total population to qualify as either endangered or threatened. For example, prior to 2001 the criteria for an Endangered assessment (Criteria A1: [35]) required a reduction of ≥50% in the total number of mature individuals over the last 10 years or 3 generations. As of 2001, this same category required a ≥70% decline [36].

The definition of full recovery that we employ here is a change in COSEWIC assessment status to ‘not at risk.’ Although this may not equate to a full ecological recovery (e.g. [37], [38]) it allows us to broadly assess trends with a consistent metric.

### Data collection

We identified all species that COSEWIC has assessed more than once using the Wildlife Species Search Engine on the COSEWIC website [33]. Assessments occurred between 1977 (COSEWIC's establishment) and December 2013 (when we conducted our search). We recognize that our analysis may be limited by biases associated with which species have been assessed multiple times and that COSEWIC listing criteria have changed since 2001. However, because the 2001 revision tightened criteria for more-severe listings, the expected bias introduced by the criteria change would be a greater proportion of less severe species listings. We used the ‘change in status’ tab on the website to include any species ‘in a higher risk category’ (N = 81); ‘in a lower risk category’ (N = 36); ‘no longer at risk’ (N = 21); ‘changed’ (N = 20); ‘reassigned’ (N = 69); and ‘no change’ (N = 272); populating our database with a total of 499 species. In our analysis, we excluded any species that had ever been assessed as ‘data deficient’ by COSEWIC (N = 30 species), or that had experienced some form of reassignment (e.g. a species split into multiple designatable units, N = 106 species), because changes in status for these species cannot be interpreted as a true change in extinction risk. We excluded six species that were both data deficient and reclassified, leaving a total of 369 species in our analysis.

We collected the history of COSEWIC assessments for each species on its respective species summary page. We recorded the date and status designation of each COSEWIC assessment for each species, and the taxonomic group to which that species belongs (due to small sample sizes, we combined molluscs and arthropods into a single ‘invertebrates’ category, and combined lichens and mosses). We then used the SARA public registry [34] to record whether species were SARA listed, and to access COSEWIC status reports for each species. To account for differences between species' life histories, COSEWIC and the IUCN place rates of decline into a biological context by scaling assessments by the generation time (GT) of each species [35], [39]. Therefore we also recorded each species' generation time, if available. If the species was SARA-listed as extirpated, endangered, or threatened, we recorded whether a recovery strategy had been completed. If the recovery strategy was completed, we recorded whether the strategy indicated that critical habitat had been fully, partially, or not identified. If a recovery strategy was not complete for the species, we recorded that critical habitat had not been identified. For SARA-listed species, Environment Canada provided us with the dates when species were listed.

For some species, the COSEWIC summary explained that an apparent improvement in status was due to the discovery of new populations through increased sampling, and not because of an actual recovery in population. We made note of these cases (N = 20) so as to distinguish them from improvements due to conservation action.

### Data analysis

We assigned each COSEWIC status a numerical value in descending order of severity (5 = not at risk, 4 = special concern, 3 = threatened, 2 = endangered, 1 = extirpated). We then calculated the overall change for each species, between the first and most recent COSEWIC assessment. For example, a species that was initially classified as special concern, and then deteriorated to endangered in its most recent assessment would receive a score of −2. For species requiring critical habitat designation (extirpated, endangered and threatened), we performed a Pearson's chi-squared test to determine whether critical habitat identifications (full, partial, or not identified) across the ten taxonomic groups differed from the proportions across all species. We used the statistical software R for computations and data plots [40].

We identified species whose most recent COSEWIC assessment occurred after the species had been listed under SARA for at least three of that species' GT, to identify species for which some recovery may be biologically possible. We then employed a cumulative link mixed model using the “ordinal” package in R [41] to test whether the number of GTs since SARA-listing was associated with a change in final COSEWIC status across species. Cumulative link mixed models test the influence of one or more independent variables (herein: number of generation times since listing) on an ordinal dependent variable (change in COSEWIC status), and are analogous to generalized linear mixed effects models in that they allow for the incorporation of a random effect, or ‘grouping’ factor (taxonomic order) [41]–[43].

## Results

**Figure 1:**
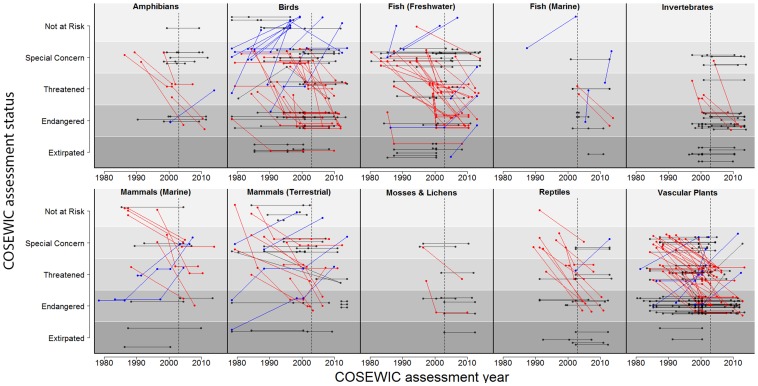
Overview of COSEWIC assessment statuses for all species that have been assessed more than once, and that have never been data deficient or taxonomically reassigned. Each species is represented by points (assessment date and outcome) connected by lines. Species that deteriorated in status from their first to final COSEWIC assessments are red, species that have improved are blue, and species that have remained constant are black. The vertical dotted line indicates 2003, or the passing of SARA. Species whose apparent recovery was due to increased sampling effort, and not biological recovery (N = 20) are not shown.

**Figure 2:**
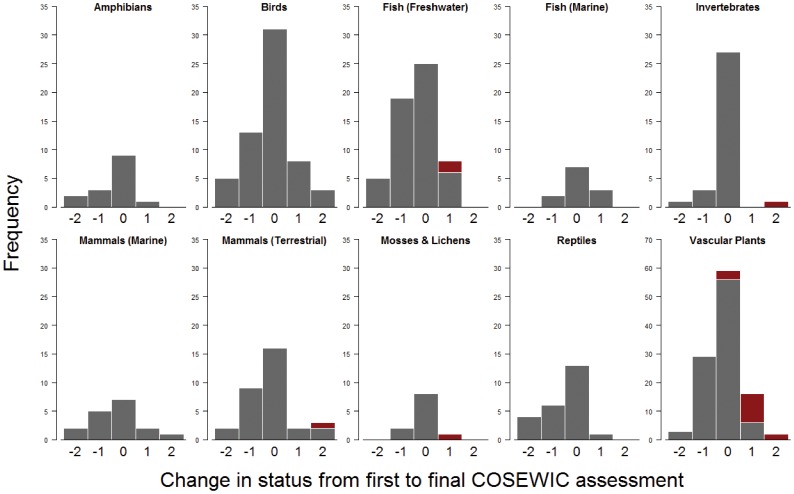
Frequencies of occurrence of change in COSEWIC assessed status for each taxonomic group. Positive numbers indicate improvement (e.g. a transition from endangered to threatened would be +1, while endangered to special concern would be +2), negative numbers indicate deterioration, and zero indicates no change across assessments. Red bars indicate apparent recoveries due to increased sampling efforts. Note that the y-axis for vascular plants is scaled differently from other taxonomic groups.

**Figure 3:**
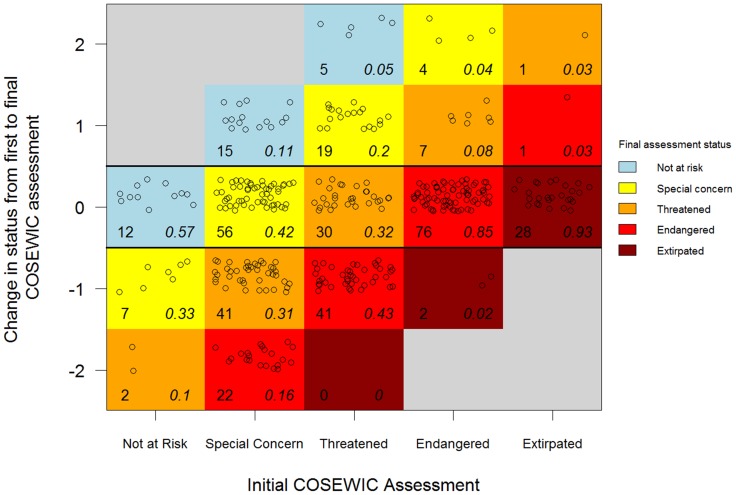
Trends in COSEWIC statuses for each species, grouped by initial assessments. Proportions are grouped by initial assessment status (column). Change from first to final assessment is indicated on the Y axis, and colours indicate the final assessment status. For example, of species that were initially assessed as threatened (third column), 19 of them (or a proportion of 0.2) improved by one status level (+1 on Y-axis), ultimately placing them into the special concern category (yellow).

**Figure 4:**
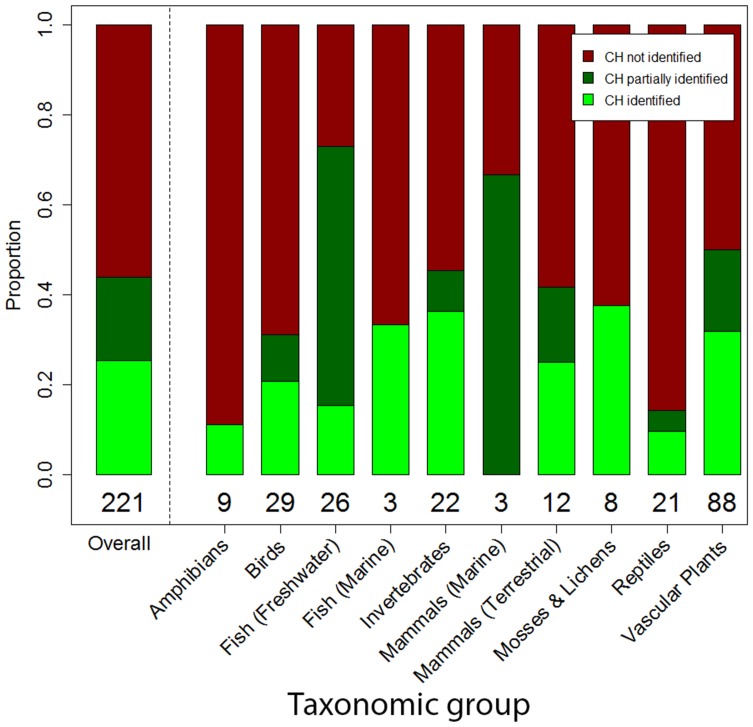
Proportion of SARA-listed species (listed as extirpated, endangered, or threatened) that have critical habitat (CH) fully identified (light green), partially identified (dark green), or not at all identified (dark red), overall and by taxonomic group. Values under bars indicate the number of species in each taxonomic category.

There were a total of 369 species across ten taxonomic groups that met our criteria for inclusion (Table S1, Fig. 1). For all taxonomic groups (except marine fish), the majority of species declined or remained the same in status (Table S1, Fig. 2). However, species trajectories varied substantially based on their initial assessment (Fig. 3). For species initially classified as not at risk, endangered, or extirpated, the most common outcome was to remain at their initially assessed status. However, for species initially classified as special concern or threatened, deterioration in status was the most common outcome. For example, 47% of species listed as special concern deteriorated in status (Fig. 3). Only 20 species (5.4%) received a ‘not at risk’ assessment after previously being listed in an at-risk category (three terrestrial mammals, nine birds, four freshwater fish, one marine fish, two vascular plants, and one lichen). Five of these cases were not due to conservation action, but were instead due to increased sampling effort. A total of 221 species are listed under SARA as threatened, endangered, or extirpated (Fig. 4), and therefore their critical habitat should be identified. Overall, and for all taxonomic groups, critical habitat has not been fully identified for more than half of SARA-listed species (Fig. 4). Further, critical habitat identifications (full, partial, or not identified) significantly varied across the ten taxonomic groups (÷^2^ = 51.90, df = 18, p<0.001).

**Figure 5:**
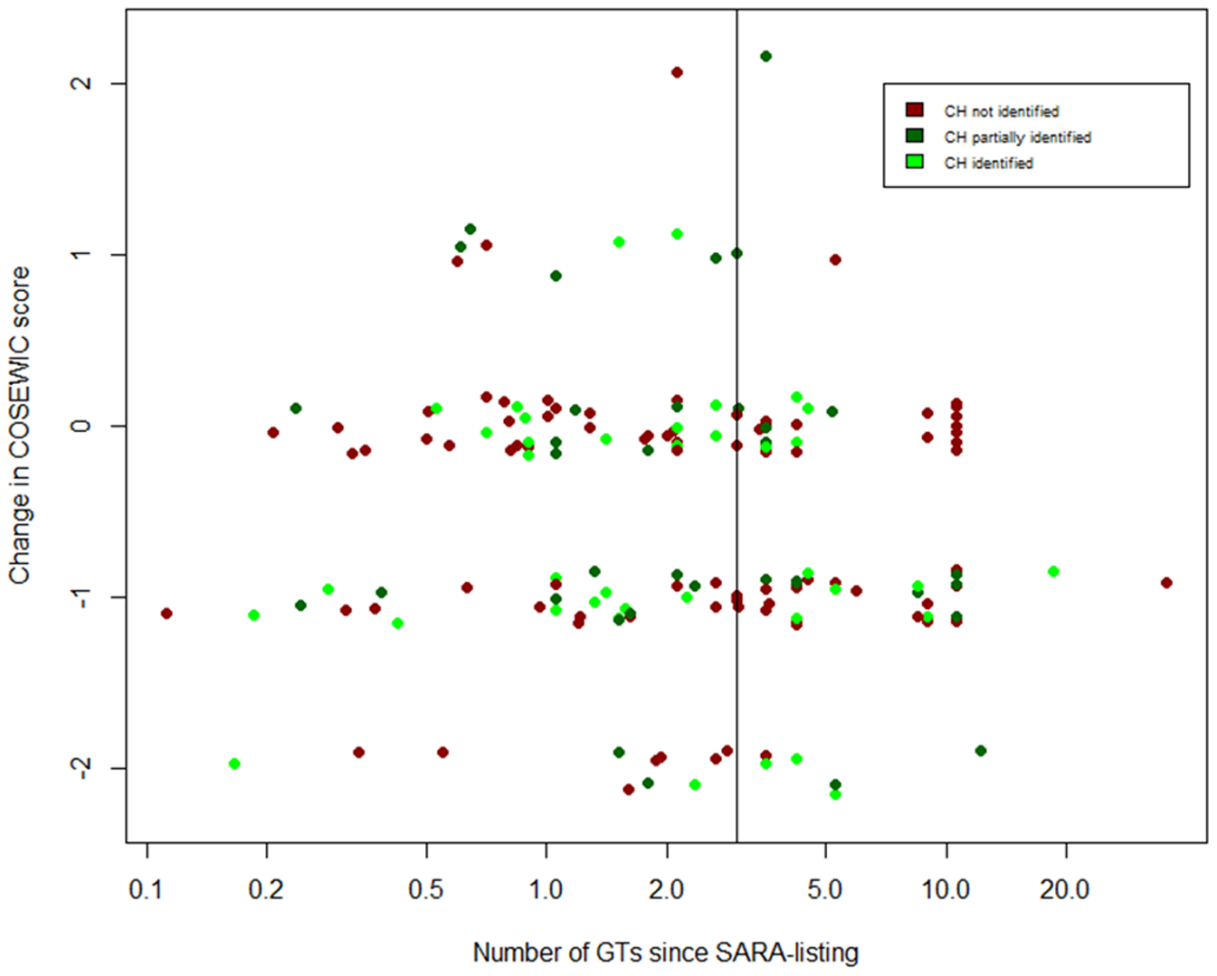
Change in COSEWIC assessment statuses versus the number of GT that have passed since initial SARA listing for all schedule 1 species listed as threatened, endangered, or extirpated. Dot colour indicates whether CH has been fully identified (green), partially identified (dark green), or not identified (red). The black vertical line indicates three GT.

There were 163 species that met our criteria for inclusion in the Generation Time analysis (listed as extirpated, endangered, or threatened; GT reported; schedule 1), and 69 that have been listed for three or more GT. The number of GT since listing ranged from 0.11 to 35.8 (mean ±1 S.D., 3.7±4.3, Figure 5). If a species has been adequately protected, the probability of a species improving in status should increase with the number of generations since listing. However, there was an estimated decline in the probability of a species improving as the number of generations since listing increased (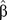 = −0.05, {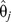} = {−2.40, −0.18, 2.44, 4.31}, 95% C.I. = −0.124 to 0.008), but it was not significant (Wald p = 0.084). This model had a small absolute gradient (7.4*10^−6^) and a condition number of the Hessian of 720, indicating that the model was able to converge and was well-defined, respectively [42]. The cumulative odds ratio was between 0.88 and 1.01 (95% C.I.), meaning that for each unit increase in GT since listing, the odds of a species improving in status ranged from increasing by 1% to decreasing by 12%.

## Discussion

For species that have been assessed more than once by COSEWIC, improvement was rare, and recovery to a ‘not at risk’ status occurred in only 5.7% of cases. Moreover, one quarter of those were apparent recoveries due to increased sampling, rather than increases in population size driven by conservation action. Contrary to the intent of endangered species legislation in Canada, the probability of a species improving in status did not increase with the number of generations since initial listing under SARA. Moreover, species had a greater probability of deteriorating in status with the number of generations since listing, although this relationship was marginally statistically non-significant (Wald p = 0.084). In contrast, species recovery in the United States was strongly correlated with the number of years of protection under the Endangered Species Act [44], indicating that endangered species legislation can be effective. These results suggest a potential failure of Canadian legislation, its subsequent implementation, or both. While COSEWIC's assessment criteria were modified in 2001 [35], the revisions made it harder to qualify for more-severe categories. Therefore we have no evidence to suggest that observed declines in status were an artifact of changes in criteria.

The lack of observed recovery for SARA-listed species may be due to the lack of implementation of the law. For example, for those species without identified critical habitat, the habitat protection provisions of SARA cannot be fully implemented. Overall, the proportion of species with critical habitat identified was higher than what has been reported in a previous study [25], although our study focused on a subset of species that have been assessed multiple times, and that have never been data deficient. These are the species for which it should have been most feasible to identify critical habitat, as they have been scrutinized for a relatively long time and have sufficient data to complete an accurate assessment. The fact remains that despite the importance of critical habitat identification, the proportion of species whose critical habitat was fully identified remained low, and varied significantly across the ten taxonomic groups. This pattern suggests that there are considerable differences between the protections that species should receive under SARA, and what is actually achieved.

Since our data demonstrate that it is rare for at-risk species to recover in Canada, it is essential that substantial efforts be made to prevent species from becoming at-risk in the first place. Given that imperiled species are usually threatened by loss of habitat [45], recent weakening of federal laws that protect habitat in Canada [25], [46], [47], [48] will be unhelpful in the long term, as it may result in additional species declining to the point where they receive an at-risk designation. Habitat protection should not be limited to critical habitat – it should be managed appropriately for all habitat such that protection does not become critical. The experience in the United States demonstrates that protection of critical habitat is associated with improved conservation outcomes relative to species without such protection [44], [47]. In addition, since we found that the most common outcome for a species assessed as special concern was to deteriorate, an assessment of special concern does not currently result in sufficient protection to promote recovery.

The single most common outcome across taxonomic groups was for species to remain at the same status across assessments, and the number of species that declined outnumbered those that recovered by a ratio of approximately 2∶1. These findings are alarming for three reasons. First, by definition, a species that has been assessed as anything other than ‘not at risk’ is at elevated risk of extinction or extirpation given current conditions, and therefore maintaining a species at a threatened status should not be interpreted as a conservation victory. Second, it takes a substantial decline in population size or range size to trigger a change in assessment status, so real declines (or increases) could still occur within species held at the same COSEWIC threat level across assessments. Third, a ‘not at risk’ designation only means that the species is not at elevated risk of extinction or extirpation – it does not imply that the population has recovered to historical levels. Even species that are classified ‘not at risk’ can be heavily depleted and unable to serve their historic roles in ecosystem structure or function. This has implications for managers in the United States as well as Canada, because many at-risk species have ranges that extend into both countries. At the very least, a successful species at risk program should demonstrate species recovering to a point where they are not at risk of extinction or extirpation given current conditions. Currently, this goal is not being achieved in Canada for the overwhelming majority of species.

## Recommendations

Our results lead us to make three core recommendations for at-risk species in Canada. First, given that it was much more common for species of special concern to deteriorate than to improve, we should recognize that a special concern listing warns of a coming deterioration, and we therefore suggest that the protections associated with this listing should be strengthened. Second, given the poor outcomes of at-risk species in Canada, it should be a policy priority to prevent species from becoming at-risk in the first place. The importance of critical habitat indicates that future legislation should be underpinned by a strong mandate to conserve habitat and we recommend that any legislative changes that may reduce habitat protection (e.g. the Fisheries Act [48]) should be reconsidered. Third, to experience conservation benefits from SARA, this law must be fully implemented. Implementation requires that critical habitat be fully identified and subsequently protected for SARA-listed species. The federal government should also be prepared to enact its ‘safety net’ provision, in the event that species in these regions are not receiving adequate protection to enable recovery (as it did with greater sage-grouse, *Centrocercus urophasianus* [49]).

Finally, even if these recommendations were accepted and put into effect, recovery takes time. Effective management requires that conservation measures be sustained over the long term, even if positive outcomes are not immediately observed.

## Supporting Information

Table S1 **Summary counts of species across taxonomic groups, for all species assessed more than once by COSEWIC.** Values for species listed for >3 generation times (GT) refer to the subset of species for which GTs were reported.
(DOCX)

Data S1 **Raw data for the present study in both wide and long formats.**
(ZIP)
