# Supplementary material for: Trends in Extinction Risk for Imperiled Species in Canada
Source: PLoS One. 2014 Nov 17;9(11):e113118. doi: 10.1371/journal.pone.0113118 (PMC4234741; doi:10.1371/journal.pone.0113118)
Supplement: Table S1 — Summary counts of species across taxonomic groups, for all species assessed more than once by COSEWIC. Values for species listed for >3 generation times (GT) refer to the subset of species for which GTs were reported. (DOCX) [file pone.0113118.s001.docx]

Table S1: Summary counts of species across taxonomic groups, for all species assessed more than once by COSEWIC. Values for species listed for >3 generation times (GT) refer to the subset of species for which GTs were reported.

| Taxonomic Group | Species (N) | # deteriorated | # stayed constant | # improved | Proportion of species with SARA-listing | SARA-listed species with reported GT (N) | SARA-listed species (excluding special concern) with reported GT | # of species listed (no special concern) for >3 GT |
| --- | --- | --- | --- | --- | --- | --- | --- | --- |
| Amphibians | 15 | 5 | 9 | 1 | 0.93 | 12 | 7 | 3 |
| Birds | 60 | 18 | 31 | 11 | 0.68 | 34 | 23 | 13 |
| Fish (freshwater) | 57 | 24 | 25 | 8 | 0.75 | 42 | 25 | 11 |
| Fish (marine) | 12 | 2 | 7 | 3 | 0.33 | 4 | 3 | 0 |
| Invertebrates | 32 | 4 | 27 | 1 | 0.97 | 13 | 8 | 3 |
| Mammals (marine) | 17 | 7 | 7 | 3 | 0.47 | 8 | 3 | 0 |
| Mammals (terrestrial) | 32 | 11 | 16 | 5 | 0.63 | 18 | 11 | 8 |
| Mosses & lichens | 11 | 2 | 8 | 1 | 0.91 | 3 | 3 | 1 |
| Reptiles | 24 | 10 | 13 | 1 | 1.00 | 24 | 21 | 1 |
| Vascular Plants | 109 | 32 | 59 | 18 | 0.98 | 68 | 60 | 29 |
| Overall | 369 | 115 | 202 | 52 | 0.90 | 226 | 164 | 69 |
